# Retracted: A Simple HPLC-UV Method for the Determination of Glutathione in PC-12 Cells

**DOI:** 10.1155/2022/9781919

**Published:** 2022-02-24

**Authors:** Scientifica

*Scientifica* has retracted the article titled “A Simple HPLC-UV Method for the Determination of Glutathione in PC-12 Cells” [1], due to concerns with data permissions. The listed authors did not collect the data presented in the article and did not possess the necessary permissions for publication of the data. It was found that the data were collected and owned by Ganesh K. Sittampalli and colleagues, and the article is therefore retracted from the journal with the agreement of the editorial board. The authors agree to the retraction.
